# Introductory Address to the 24th Annual Session of the Ohio College of Dental Surgery

**Published:** 1869-11

**Authors:** A. M. Moore


### DENTAL REGISTER.

Vol. XXIII.] NOVEMBER, 1869. [No. 11.

### Original Communications.

### INTRODUCTORY ADDRESS TO THE 24th ANNUAL SESSION OF THE OHIO COLLEGE OF DENTAL SURGERY.

BY PROF. A. M. MOORE.

Gentlemen :—Called by the Board of Trustees of the Ohio College of Dental Surgery to fill the chair of Clinical Dentistry in the corps of teachers, I appear before you this evening at the request of my colleagues to address you on the occasion of this, the opening of the 24th course of lectures in the College.

Unused to public speaking, I shall throw myself on your kind indulgence, while, for a short time, I call your attention to a few thoughts hastily thrown together, and deemed of importance and worthy our consideration at this time.

But, before I proceed, permit me to call your attention to a brief view of our position, past and present, and compare it with its surroundings.

It is healthful at all times, and in all the circumstances of life, to review the past, whether the cause has been progression or retrogression. If the first, it inspires a spirit of determination to make still farther and higher efforts; if the latter, it will beget a spirit of regret for the past, and a zeal for the future, that shall atone for its neglects.

A quarter of a century ago, and Cincinnati was not what she is to-day, in any respect we may view her. Her borders were limited, her commerce restricted, her merchants contracted in their means. The canal and river, with comparatively few and small boats carried all the produce of her merchants and manufacturers; and as in these respects, so in all others, whether of education, science or the arts. How is it to-day? Her borders are enlarged to such an extent that it is seriously contemplated to bound them only by the principal lines of the county, while her population has increased to a fabulous extent. Her commerce is scattered among all the nations of the earth, and all parts of the world are made tributary to her prosperity. Her merchants are merchant princes, her rivers are covered with boats mammoth in size, and adapted to the wants of commerce and travel. Her railroads enter from all directions ladened with the rich productions of the soil. The busy hum of industry and manufacture, the fostering care of education in the extensive schools and colleges and beautiful churches; her observatories, her libraries, art and literary associations, conspire to make her, indeed, the "Queen City of the West." She gives liberally of her means for the development of the resources of the country around her. She extends her arms to embrace the whole continent, by offering to give ten millions for a railroad to Charleston and the South, and as much more for a direct way to Norfolk and the East. The lone ferry-boat has given place to the proudest achievement of modern times, the architectural skill, beauty and workmanship of which commands the respect of the world. It was in this early period of which we speak, in the history of this great city, that it entered the minds of a Taylor, the Allens, a Cook, and a few other kindred spirits to organize here an institution to elevate the standard of Dental education in the West. It was an experiment fraught with interest and hazardous in its nature. There was, at the time, one college located in the East, and to sustain another, though being located in the West, was a matter of great solicitude with its founders; but they counted the cost, and having put their hands to the plow determined not to falter, and thus this college edifice was erected at a heavy expense to its founders.

The twenty-three classes of graduates who have scattered abroad over the world, and this meeting to-night, all attest the wisdom and energy of its founders. And here, to-night, in the person of your honored Emeritus Professor and President of the Board, stands the one who yet guards and helps to sustain that which his own hands helped to form, and his wisdom to conceive. And, if this institution, with its present enviable reputation, with which he is honorably connected is not honor enough, then we grant him the higher honor of first conceiving and publishing the idea of the Atlantic telegraph.

In the year 1845, the year in which this college was founded, Prof. Taylor, in an address before the American Society of Dental Surgeons, in the city of New York, made use of the following remarkable language:

"In what an age we live! The sluggish onward trot of steam's propelling power suits not this go ahead generation. But with the lightning's flash they press on to greatness, beneath, above, around, and pressing in at every pore, an agent stands ready for our bidding. As quick as thought, it carries thought through illimitable space, and reports with truthful fidelity every word and sentence. With one foot on the western shore of the Atlantic, while the other ready to stride the mighty ocean, and land with the latest news instanter in London. Scarce can you count the ticks which make up a moment of time, before the circuit of the earth is done."

If that is not the Atlantic telegraph forshadowed, it is not now in operation.

Of such men were the early founders of this college. And as they may have passed away either from active duties of professorship or board of control, their mantles have fallen on men of like spirit with whom success was the only object, aim and determination.

To-day the Ohio College of Dental Surgery, under the control of the several Boards of Trustees who have from time to time controlled its destinies, and under the auspices of its body of stockholders who have pledged themselves and their stock on the altar of its success, stands forth among the several Dental institutions of the country as second to none.

The object of this College being the elevation and advancement of our specialty, let us inquire by what means this can best be accomplished. And here let me remark, that the medical profession is proverbial for its warlike propensities arising from the fact, that while most other professions can settle their differences by reference to books of standard reputation in their profession, it is one solely of opinion, founded on symptoms very nearly resembling each other, and in many cases entirely impossible to draw the line of difference until death. This doubtless, is the cause of most if not all the serious difficulties which arise in the medical profession, and each has a right to contend for the correctness of his diagnosis until ultimately and surely settled, (life and death being the issue) each has a right to assert his as the most *correct.*

Notwithstanding our specialty is closely allied to medicine, we have marked symptoms, and may, if we are skillful, diagnose with mathematical precision. For this reason harmony should pervade our ranks. Therefore, to attain perfect success, there must be first a perfect harmony between the Board of Trustees and the entire corps of teachers in all that affects the interests of the College.

The Board of Trustees is composed of gentlemen members of the profession and eminent as such, from four different sta es, all interested as stockholders, and zealous for the good name of the College, and the dignity and success of the profession. These gentlemen, thus circumstanced have, with perfect unanimity, adopted a course of study full and comprehensive, and have called to fill the several chairs (with the exception of your speaker), gentlemen known and acknowledged as among the most worthy and successful teachers.

This great object having been so happily attained in the wisdom of the Board, their intercourse with one another should be harmonious and peaceful. They should be kindly affectionate one to another—in honor preferring one another.

In the manifest wisdom of the Board, the chairs have been so amicably arranged that in teaching each one is independent of the other, and, at the same time depending one upon the other. Like the organs of the human body they form a perfect whole ; yet take away any one, and the harmony and perfection of the machinery is destroyed. These dependencies and courtesies strictly observed, our intercourse will be pleasant in the present, and happy in the retrospection ; our efforts will be rewarded with rich success, and all, whether board, teacher, stockholder, student, or friend will rejoice in the happy fact that circumstances and destiny had united our fortunes.

A second means of success in our enterprise is, that we must cultivate a generous intercourse with the medical profession, whether in their colleges, or in the ordinary walks of private practice.

It is in the memory of many of us, when the arrogant disciple of Esculapius was wont to thrust his tongue in his cheek in derision of our new-born profession. But those times are happily past, and the days of charlatanry are rapidly passing away from our view. We stand forth to-day, a profession of acknowledged merit, the equal in everything that renders either branch worthy the favor or regard of the other.

The days of specialties are fully upon us. Surgery, medicine and obstetrics are all branches of the one, and Dental surgery is acknowledged by all as a distinct branch of the same.

Some of the most brilliant feats of modern surgery owe their perfect and entire success alone to the aid of our art. These several branches have now become co-workers in the great work of alleviating the sufferings of mankind, each in its legitimate sphere performing an important part, without which, success would be regarded more doubtful and uncertain. This being the case, how important it is that there be full and complete harmony between the gentlemen who fill the several ranks of the different branches of the *one* great profession.

The third means of obtaining success in our undertaking is by a proper interest in, and intercourse with the students. No two individuals in the class have the same cast of mind, the same habits, the same physical developments. Coming from different sections of our country, each brings with him his peculiar habits and manners. These different classes must be treated with respect by their teachers; not sustaining them in any habits which should be condemned, but winning their confidence and esteem by giving good and wholesome advice. It sometimes happens, that a student who first seems dull and plodding, calculated to weary the patience of his teachers suddenly emerges from his dullness, and becomes one of the brightest ornaments of his profession. A mother's approving kiss made West all he ever attained as an artist. And instances are not rare, in the reading of most of us, where unkindness and cruelty smothered real genius. While one kind word struck a spark, that in its bright effulgence dazzled the world of science and art.

Our intercourse with them must, therefore be pleasant. Our labor profitable to them and agreeable to ourselves, and when the time transpires, and they are to separate from these hallowed walls, they may be a blessing to their adopted community, an honor to themselves, and a lasting glory to their *Alma Mater*.

Finally, gentlemen of the Board, our library and museum must be enlarged, and filled with all that can possibly tend to their more extended usefulness. Our means of instruction must be greatly increased. Our building enlarged to meet the increasing necessities. Our chairs of instruction divided and increased, that in each branch the utmost means of instruction, theoretical and practical may be given. There is no standing still! We are now in the front ranks! The mighty tide of universal progress comes rolling behind us. Onward we must proceed.

I have thus far, in accordance with the design of this address, spoken of matters and things in general—of the glorious progress and present condition of our profession as an art, and a science, let me now, in conclusion, speak to the young men entering this College.

It would be absurd in me to ask what has brought you here? The place in which we are assembled is a sufficient answer. The object is to prosecute your studies under new auspices. To avail yourselves of new means and opportunities for extending and perfecting the knowledge you have brought with you. Many of you never were in a Dental college, never performed a dissection, nor witnessed a chemical experiment. You enter now upon a new life. The office of a Dentist, with here and there an exception, offers comparatively few facilities for the acquisition of a knowledge of a science so vast as that of ours. In a well conducted course of lectures more may be learned in a single month than in the two years ordinarily spent in the private office. There are numberless principles in anatomy, chemistry, materia medica, and metallurgy which can only be taught by an appeal to demonstration such as no private office can supply.

To-morrow morning, bright and early, you will buckle on your armor of industry. Begin your work with earnestness, like men determined. Surmount all difficulties—fulfill the great mission as honest dilligent students. At first the way may seem difficult—your arduous duties will weary and perplex you, but day by day your progress will become easy and thus step by step you will steadily mount up higher and higher the steep hill of science. When, at length, standing upon its lofty summit, you may leisurely and calmly survey the majestic scenery around and beneath you, with the proud consciousness of having done your duty.

We your teachers too, who, like yourselves, once sat upon hard, backless benches in quest of knowledge. We too, now, will be students with you. With our torch in hand we will accompany you in all your journeyings, point out the way, assist in removing obstacles, and thus, working and laboring together, share your toils and pleasures.

Although it will be the duty of each of my colleagues to inform you of the best method of studying his own particular department, yet I can not refrain from referring in a few general terms to the subjects which will especially engage your attention during the brief session upon which you have entered.

To anatomy you should give special attention, not learning from books alone, but from nature; tracing out, scalpel in hand, every important structure, normal and abnormal, true and false, and thoroughly photographing it upon the mind; and in carrying out your dissections you will often be led to realize the truth and force of the words of that man of patience, "Man how fearfully and wonderfully made;" or that other and more poetical, "Strange that a harp of a thousand strings can keep in tune so long."

Chemistry, one of the elementary branches, can only, as your able professor will himself inform you, be learned in the laboratory; like anatomy, which can only be learned in the dissecting room, or operative Dentistry in the chair. While chemistry is the most abstruse and difficult of studies, it at the same time is the most sublime and fascinating—one which is better calculated to establish a closer relation between man and his Creator than perhaps any other, inasmuch as it associates him with some of his sublimest creations.

In the study of *Materia Medica*, you will learn how greatly our science is indebted to this department, for the control of diseased action, and under the guidance of your able teacher you will learn what estimate to place upon the more important articles which are used in our specialty.

Operative and Mechanical Dentistry are so closely allied, so intimately interwoven, that they constantly trench one upon the other. Some of you may make good operative Dentists—possess all that fine manipulative skill and superior judgment that crowns the skillful Dentist with his success, and not attain to the highest degree in Mechanical Dentistry, and *vice versa.*

The teachers who preside over these departments will spare no pains to qualify you for the arduous and responsible duties of practitioner—the great aim of all your studies and energies.

Clinical Dentistry will constitute a special object of attention. Every opportunity will be embraced to illustrate the principles enunciated in the didactic course. The clinics of this institute are proverbial for their numbers and variety of material, and you can not, if you give it your proper attention but be benefited.

I have said nothing thus far of the studies of physiology and pathology. In these you have a ready teacher, and interesting studies. These branches will constantly lead you step by step on and up, and with anatomy and chemistry will furnish you a ready key to their interesting and seductive branches of Dental science.

Thus, gentlemen, you see what is before you. Remember, "success can only be securely built upon the solid block cut from the quarries of truth and science." "*Nil sine suaore*" is the motto. Work then systematically and steadily. Fidelity to the cause you have espoused is your first and important duty. Honor it then; exult in its origin, its dignity and grandeur—in its power of doing good. The knowledge you acquire while within these walls is your capital stock upon which you will realize in proportion as it is appreciated in the market in which you operate. Par value is good, but to be profitable it must give you a margin.

It has been sneeringly said that a young man who is good for nothing else is good enough to be a Dentist. There never was a more erroneous assertion. In none of the learned professions is a higher grade of talent and mental culture required than in ours. The fact that all Dentists are not greater is no proof that they are destitute as a body of talent and genius.

Distinction great and permanent, such as elevates the character of a nation, is the prerogative of the few, and not of the many, and measured by this standard the Dental profession has just cause to be proud of its exalted position in the scale of intellect and true greatness. Mankind have little idea of the knowledge that is required to successfully perform the multifarious operations coming within the proper jurisdiction of the Dental operator.

They lose sight of the fact that the human body is a living machine composed of numerous organs and tissues, each invested with important functions, and all united by the closest and most intimate relationship.

Finally, gentlemen, in conclusion, let me say to you, spend your time wisely when without these walls. "Remember the Sabbath day to keep it Holy." Be strong in your resolves to withstand temptation. Do not disregard the admonitions of parental affection and friendship. The last words of a fond and doting mother whispered into your ear, as she sobbingly pressed your hand, and imprinted her last kiss. "My son, shun the ways of evil, and neglect not the church and your Bible."

